# The Emerging Roles of mTORC1 in Macromanaging Autophagy

**DOI:** 10.3390/cancers11101422

**Published:** 2019-09-24

**Authors:** Akpedje S. Dossou, Alakananda Basu

**Affiliations:** Department of Microbiology, Immunology and Genetics, University of North Texas Health Science Center, Fort Worth, TX 76107, USA; Akpedje.Dossou@my.unthsc.edu

**Keywords:** macroautophagy, autophagy regulation, mTORC1 substrates, AMPK, ULK1, autophagy initiation, nucleation, elongation, autophagosome maturation, transcriptional regulation

## Abstract

Autophagy is a process of self-degradation that enables the cell to survive when faced with starvation or stressful conditions. The mechanistic target of rapamycin (mTOR), also known as the mammalian target of rapamycin, plays a critical role in maintaining a balance between cellular anabolism and catabolism. mTOR complex 1 (mTORC1) was unveiled as a master regulator of autophagy since inhibition of mTORC1 was required to initiate the autophagy process. Evidence has emerged in recent years to indicate that mTORC1 also directly regulates the subsequent steps of the autophagy process, including the nucleation, autophagosome elongation, autophagosome maturation and termination. By phosphorylating select protein targets of the autophagy core machinery and/or their regulators, mTORC1 can alter their functions, increase their proteasomal degradation or modulate their acetylation status, which is a key switch of the autophagy process. Moreover, it phosphorylates and alters the subcellular localization of transcription factors to suppress the expression of genes needed for autophagosome formation and lysosome biogenesis. The purpose of this review article is to critically analyze current literatures to provide an integrated view of how mTORC1 regulates various steps of the autophagy process.

## 1. Introduction

Autophagy is a catabolic, or literally a “self-eating”, cellular process whereby macromolecules or organelles are degraded in the lysosomes for nutrient recycling to cope with cellular stress and nutrient shortage [1]. Based on the way targeted molecules or organelles are taken up by the lysosomes, autophagy can be classified as macroautophagy, chaperone-mediated autophagy (CMA) or microautophagy. Macroautophagy features the encasement of the cytosolic materials to be degraded in a double-membrane vesicle termed autophagosome. The loaded autophagosome, then fuses with a lysosome for degradation of the autophagosome’s cargo and some of its constituents [2]. While macroautophagy is non-selective bulk degradation of cellular components, CMA is more of a selective process whereby non-functional, damaged soluble proteins are recognized by a chaperone, and translocated into the lysosomal lumen for degradation [3,4]. In microautophagy, the cytosolic content is directly engulfed by the lysosome through lysosomal invagination, lysosomal protrusion or endosomal invagination [5].

**Figure 1:**
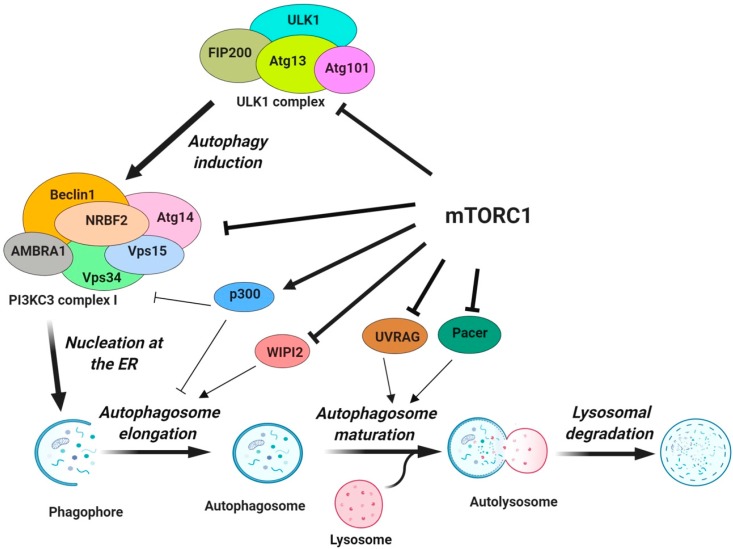
Regulation of various steps of autophagy by mTORC1. mTORC1 inhibits the activity of the ULK1 complex by phosphorylating ULK1 and Atg13. The nucleation step of autophagy is inhibited via the phosphorylation of Atg14, AMBRA1 and NRBF2 in the PI3KC3 complex I. Phosphorylation of p300 and WIPI2 by mTORC1 inhibits VSP34 activity/LC3 lipidation and the recruitment of phosphatidylinositol phosphates along with the LC3 conjugation system for the autophagosome elongation. Finally, mTORC1 negatively regulates the fusion of the autophagosome with the lysosome through the phosphorylation of UVRAG and Pacer that are important for the lipid kinase activity of PI3KC3 complex II and the recruitment of the HOPS tethering complex. The image was created with BioRender.com.

Macroautophagy (henceforth referred to as autophagy) has been studied most extensively. The process starts with the activation of the induction complex, which recruits autophagy-related (Atg) proteins for the nucleation process (Figure 1). The nucleation of the isolation membrane at the endoplasmic reticulum (ER) forms the cup-shaped phagophore [6]. Subsequently, the phagophore is extended into the double-membrane autophagosome, which once sealed will include cytosolic materials to be degraded. Then, the autophagosome traffics to lysosome to complete the step known as autophagosome maturation whereby the autophagosome fuses with the lysosome. The formation of the autolysosome followed by the degradation of the inner membrane constituents of the autophagosome and cytosolic materials through lysosomal enzymes concludes the autophagic flux [7].

The bulk of the steps involved in autophagy and associated regulatory mechanisms are evolutionarily conserved among eukaryotic cells, albeit the level of complexity of these processes may differ [8]. This relative conservation of mechanisms is also observed with Tor (target of rapamycin) proteins, which play critical roles in nutrient sensing, cellular metabolism and growth [9,10]. Both autophagy and Tor signaling have been studied extensively in yeast. Genetic screening in yeast *Saccharomyces cerevisiae* by Ohsumi and colleagues identified several mutants defective in autophagy, including *Apg I* (autophagy) [11], which was shown to encode for a Ser/Thr protein kinase homologous to Unc-51 protein kinase in *Caenorhabditis elegans* and was named Apg1p [12]. The activity of Apg1p was induced by starvation as well as by the Tor inhibitor rapamycin [13], which mimicked the effect of starvation in inducing autophagy in nutrient-rich condition, thus establishing a link between Tor signaling and autophagy. Once unified nomenclature was adapted, Apg1 was replaced by Atg1 (autophagy-related) [14,15]. In mammals, there are several homologues of Atg1. ULK (Unc-51-like kinase)-1 and -2 are most closely related and have been associated with autophagy but most of the studies have focused on ULK1 [16]. The mammalian homologue of Tor is referred to as mTOR (mechanistic/mammalian target of rapamycin).

mTOR is now recognized as the master regulator of autophagy [17,18]. Although mTOR was first shown to negatively regulate autophagy by inhibiting the induction step, recent studies show its involvement much beyond this initial step. We believe a comprehensive review of how mTOR regulates various steps of autophagy in a single article should be of interests to the autophagy aficionados.

## 2. mTOR Complexes: One Kinase, Two Distinct Complexes

mTOR is a serine/threonine kinase that belongs to the phosphatidylinositol 3-kinase (PI3K)-related kinase family [18,19]. It forms two complexes mTOR complex 1 (mTORC1) and mTOR complex 2 (mTORC2) with distinct composition and function [20]. The most distinguishing feature of these two complexes is the binding of the adaptor proteins, the regulatory-associated protein of mTOR (Raptor) and the rapamycin-insensitive companion of mTOR (Rictor) to mTORC1 and mTORC2, respectively. While both mTORC1 and mTORC2 bind the mammalian lethal with SEC13 protein 8)/G protein beta subunit-like (mLST8/GβL) and the DEP domain-containing mTOR-interacting protein (DEPTOR) [18], mTORC1 contains the proline-rich Akt1 substrate of 40 kDa (PRAS40) whereas mTORC2 associates with the mammalian ortholog of SAPK interacting protein 1 (mSin1) and the protein observed with Rictor 1 and 2 (Protor 1/2) [21,22,23].

mTORC1 is activated by the Ras family of GTP binding proteins, such as ras homolog enriched in brain (Rheb) [18]. Rheb interacts with mTORC1 in GTP bound state and activates it. Rheb is inactive in its GDP bound form due to interaction with the TSC (tuberous sclerosis complex)-1 and -2, which function as a GTPase-activating protein (GAP). In response to growth factors, the PI3K/Akt pathway or the Ras/Raf/ERK pathway become activated and phosphorylates TSC1/TSC2 to inhibit it. Rheb is then released from the complex, becomes activated by exchanging GTP for GDP and activates mTORC1 [24,25]. Amino acids can activate mTORC1 via a TSC1/2 –independent pathway involving Ras-related GTPases (Rags) and the guanine-exchange factor Ragulator [26]. When amino acids are abundant, the vacuolar H+ ATPase (V-ATPase) senses the rise in amino acids and activates the Ragulator, which loads Rag A/B with GTP and Rag C/D with GDP. The heterodimer formed by Rag A/B and Rag C/D recruits mTORC1 via Raptor to the lysosome where mTORC1 is activated by the lysosome-associated Rheb [26,27]. The activity of mTORC1 is also regulated by the AMP-activated protein kinase (AMPK), which senses the energy status of the cells and is activated when energy levels drop [28]. AMPK negatively regulates mTORC1 by phosphorylating Raptor or TSC2 [29,30,31].

mTORC2 is activated primarily via the growth factor/PI3K signaling [32]. The catalytic activity of mTORC2 is inhibited due to interaction with the pleckstrin homology (PH) domain of Sin1. The activation of PI3K generates PIP3, which interacts with the Sin1-PH and relieves this autoinhibition [33]. Although mTORC1 and mTORC2 have disparate substrates, the signal transduction elicited by the complexes allows them to regulate each other. Akt, which acts upstream of mTORC1, is a substrate for mTORC2 and can also activate mTORC2 via a positive feedback loop [34]. Downregulation or inhibition of mTORC1 can cause activation of mTORC2 indirectly via insulin/PI3K pathway [18]. A recent study showed that during glucose withdrawal or energy stress, AMPK could directly interact with and activate mTORC2 independent of AMPK-mediated mTORC1 inhibition [35]. mTORC1 has been studied extensively in the context of autophagy. This is due to its critical role in sensing and integrating growth factor signaling, nutrient, redox and energy levels in the cell [25,36]. In nutrient-poor and stressful conditions, inhibition of mTORC1 triggers autophagy for survival [17,37].

## 3. Regulation of Autophagy by mTORC1

### 3.1. The Protein Core Machinery of Autophagy as a Direct Target of mTORC1

Figure 1 depicts an overview of different steps in the autophagy process regulated by mTORC1. In the early 2000s, numerous studies showed that mTORC1 impacts autophagosome biogenesis by inactivating, through phosphorylation, the autophagy regulatory complex formed by ULK1 and its interacting proteins, the autophagy-related protein 13 (Atg13) and the focal adhesion kinase family interacting protein of 200 kDa (FIP200) [38,39,40]. mTORC1 intervenes with the nucleation process of autophagy by targeting components of the class III PI3K complex I (PI3KC3-CI) necessary for the isolation membrane formation [41,42,43,44,45]. Recently, mTORC1 has been implicated in the regulation of the elongation step of the autophagosome formation and the conjugation of LC3 to the autophagosome membrane by targeting the WD repeat domain phosphoinositide-interacting protein 2 (WIPI2) and the acetyltransferase p300, respectively [46,47]. Additionally, the fusion of autophagosome to lysosome in the late stages of autophagy and the termination of autophagic flux via lysosomal tubulation are also regulated by mTORC1 via the UV radiation resistance-associated gene (UVRAG) and the protein associated with UVRAG as autophagy enhancer (Pacer) [48,49,50]. In the following sections, we have elaborated on how mTORC1 regulates each step of the autophagy process. Table 1 lists mTORC1 substrates that regulate various steps of autophagy and the sites of phosphorylation. Please note that the phosphorylation sites of mTORC1 substrates in human versus mouse may differ by one amino acid. 

#### 3.1.1. Regulation of ULK1 Complex During Autophagy Initiation 

**Figure 2:**
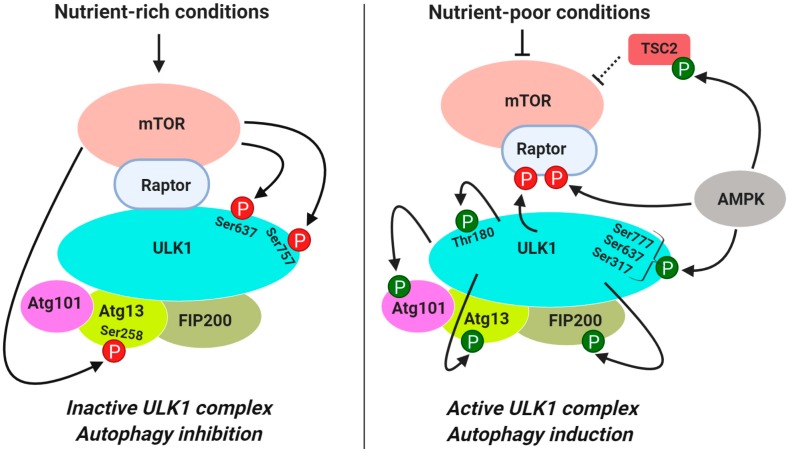
Regulation of ULK1 complex by mTORC1. mTORC1 phosphorylates ULK1 on Ser637 and Ser757 and phosphorylates Atg13 on Ser258. During starvation when mTORC1 is inhibited, the inhibitory phosphorylation is relieved by phosphatases, and the ULK1 complex becomes active by autophosphorylation at Thr180 and phosphorylates Atg13, FIP200, Atg101 and other Atg proteins. AMPK can activate ULK1 either by negatively regulating mTOC1 by phosphorylating Raptor or TSC2 or directly phosphorylating ULK1. ULK1 can also regulate mTORC1 by phosphorylating Raptor through a negative feedback loop. “P” indicates phosphorylatable residue. Red indicates negative regulation of the target proteins via phosphorylation. Green indicates positive regulation of the target proteins via phosphorylation. The image was created with BioRender.com.

In mammalian cells, the activation of the complex formed by the Ser/Thr kinase ULK1 (homolog of yeast Atg1) and its interaction partners Atg13, FIP200 (homolog of yeast Atg17) and Atg101 (gene product of C120rf44, no known yeast homolog) is critical for the induction of autophagy [51,52,53] (Figure 1). In contrast to its yeast counterpart, this complex is formed constitutively and is not affected by the nutrient status of the cells [38,39,52]. It serves as the main interface where the direct intervention of mTORC1 in autophagy occurs. In nutrient-rich conditions when mTORC1 is active, it associates with the ULK1 complex via Raptor and phosphorylates ULK1 at Ser757 and Ser637 (equivalent to human Ser758 and Ser638) [54,55], and Atg13 at Ser258 [56] (Figure 2 and Table 1). mTORC1-mediated phosphorylation of ULK1 and Atg13 inhibits the autophagy-promoting kinase activity of ULK1 complex [40]. During starvation and cellular stress when mTORC1 is inactive, it dissociates from ULK1 and the phosphorylation of ULK1 and Atg13 at the inhibitory sites is relieved by phosphatases [57,58]. While ULK1 is dephosphorylated by protein phosphatase 2A (PP2A) [57] and protein phosphatase 1D magnesium-dependent delta isoform (PPM1D) [58] Atg13 is dephosphorylated by PP2C phosphatases, Ptc2 and Ptc3 [59]. ULK1 becomes activated by autophosphorylation at Thr180 [60] and phosphorylates Atg13, FIP200 and Atg101 [40,51,61] (Figure 2). The active ULK1 complex then translocates to the isolation membrane at the endoplasmic reticulum where autophagy is initiated [51,56].

Since AMPK negatively regulates mTORC1, it is expected to positively regulate autophagy by inhibiting mTORC1. Several studies showed that AMPK directly interacts with and phosphorylates ULK1 to promote autophagy but mTORC1 influences the interaction between ULK1 and AMPK [54,55,62]. There are, however, controversies whether mTORC1 promotes or prevents AMPK/ULK1 interaction [54,55,62]. Lee et al. first reported that AMPK binds to ULK1 at the Pro/Ser-rich domain containing amino acids 654–828, and this interaction was necessary for the induction of autophagy [62]. When AMPK is activated, it associates with the ULK1-mTORC1 complex and recruits 14-3-3 binding protein to Raptor. Phosphorylation of raptor at Ser792 by AMPK inhibits mTORC1 activity, causing activation of ULK1 [62]. Bach et al. reported that phosphorylation of ULK1 at Ser555 by AMPK was responsible for the binding of ULK1 to 14-3-3, but this binding was independent of mTORC1 [60]. 

Kim et al. showed that phosphorylation of ULK1 at Ser317 and Ser777 by AMPK was associated with the induction of autophagy, and mTORC1 could inhibit autophagy by preventing ULK1/AMPK interaction [54]. It was proposed that mTORC1-mediated phosphorylation of ULK1 at Ser757 located at the AMPK binding region of ULK1 was responsible for preventing the interaction between ULK1 and AMPK [54]. In contrast, Shang et al. reported that ULK1 associates with AMPK when nutrient is available, and the complex dissociates during starvation [55]. mTOR was shown to phosphorylate ULK1 at both Ser758 and Ser638. Since mutation of Ser758 to non-phosphorylatable Ala (S758A) decreased ULK1/AMPK interaction, the authors proposed that phosphorylation of ULK1 at Ser758 was required for ULK1/AMPK interaction. Interestingly, Kim et al. also observed that the S757A mutation of ULK1 inhibited ULK1/AMPK interaction. The authors suggested that the chemistry of the hydroxyl group at Ser757 of ULK1 is critical for its interaction with AMPK since the S757C (Ser to Cys mutation) mutant retained some interaction [54]. However, this did not explain why binding of AMPK to S757C ULK1 was much less compared to wild-type ULK1 [54]. 

Several factors need to be taken into consideration to explain how interplay between mTORC1, ULK1 and AMPK regulates autophagy. First, the differences in media composition could greatly influence the experimental outcome. For example, Kim et al. observed AMPK-dependent ULK1 activation only under glucose starvation but not during amino acid starvation [54] whereas Shang et al. studied ULK1/AMPK interaction in HBSS starvation medium [55]. The media composition could also influence different steps of the autophagy, such as autophagosome formation, autolysosome formation and autophagy flux differentially. In earlier studies, autophagy was monitored by the increase in lipidated LC3-II and GFP-LC3 puncta, which could also be due to a blockade in autolysosome formation or autophagy flux [63]. A recent study showed that amino acid starvation, which caused inhibition of mTORC1 and dephosphorylation of ULK1 at Ser757 and Ser637, increased autophagy flux [64]. However, glucose starvation, which caused activation of AMPK and phosphorylation of ULK1 at Ser555, blocked autophagy flux [64]. Shang et al. also showed that when cells were starved in HBSS, both mTORC1 and AMPK phosphorylated ULK1 at Ser638 even though there is a reciprocal relationship between AMPK and mTORC1 [55]. 

Second, while the activation of mTORC1 inhibits ULK1 by phosphorylating Ser637 and Ser757, dephosphorylation of ULK1 by phosphatases is necessary for ULK1 activation. It has been reported that amino acid starvation was more effective in inducing autophagy compared to mTORC1 inhibitor rapamycin due to increase in phosphatase activity [57]. Although mutation of Ser638 (mouse Ser637) to Ala did not affect interaction of ULK1 with AMPK [55], dephosphorylation of Ser637 and not Ser737 by either the PP2A [57] or PPM1D [58] induced autophagy in response to starvation and genotoxic stress, respectively. 

Third, both mTORC1 and AMPK can regulate autophagy initiation by phosphorylating Atg13, a binding partner of ULK1. Puente et al. showed that mTORC1 directly phosphorylates Atg13 at Ser258 whereas AMPK phosphorylates it at Ser224 [56]. Dephosphorylation of both Ser258 and Ser224 on Atg13 was required for activation of ULK1 and subsequent phosphorylation of Atg13 at Ser318 by ULK1 [56], suggesting that AMPK, like mTORC1, may negatively regulate autophagy by phosphorylating Ser224 of Atg13. These results are consistent with the report by Nwadike et al. that activation of AMPK under certain conditions (e.g., glucose starvation) inhibits autophagy [64]. 

Finally, a feedback regulation between ULK1 and mTORC1 may also influence autophagy induction. It has been reported that knockdown of either ULK1 or Atg13 increased phosphorylation of S6K1, a downstream target of mTORC1, suggesting that ULK1 and Atg13 negatively regulate mTORC1 [65]. Consistent with this report, Dunlop et al. reported that overexpression of ULK1 inhibited mTORC1 signaling by phosphorylating Raptor at multiple sites, causing an interference with mTORC1 substrate recognition [66]. Consequently, knockdown of ULK1 caused activation of mTORC1 signaling as judged by increased phosphorylation of its downstream targets S6K1 and 4E-BP1 [65]. This negative regulation of mTORC1 by ULK1 may favor autophagy induction once autophagy is initiated. 

#### 3.1.2. Regulation of Vps34-beclin 1-Atg14 Complex (PI3KC3-CI) during Nucleation

While initiation of autophagy requires protein kinase activity of the ULK1 complex, the nucleation of phagophore requires the lipid kinase activity of the class III phosphatidylinositol 3-kinase (PI3KC3) complex, which generates phosphatidylinositol-3 phosphate (PI3P) from phosphatidylinositol at the phagophore. PI3KC3 forms two distinct complexes. The complex I (PI3KC3-CI) binds to Atg14 whereas complex II binds to UVRAG in a mutually exclusive fashion [67,68]. PI3KC3-CI, which consists of the catalytic subunit Vps34 (vacuolar protein sorting 34), Beclin 1, Vps15 (gene product of PIK3R4), Atg14 (also called Atg14L or Barkor), AMBRA1 (activating molecule in Beclin 1-regulated autophagy protein 1) and NRBF2 (nuclear receptor-binding factor 2), is involved in the nucleation of phagophores. Under basal conditions, the PI3KC3-CI remains associated with the cytoskeleton [69]. Once the ULK1 complex is activated, it translocates to the sub-domains of the endoplasmic reticulum (ER) called omegasomes and recruits the PI3KC3-CI to produce PI3P at the phagophore to enable the nucleation of autophagosomes [44,53]. Activation of ULK1 complex or inhibition of mTORC1 results in phosphorylation of Beclin 1 at Ser15 and Ser30 causing activation of the PI3KC3-CI [70,71]. mTORC1 can also directly regulate the activity of the PI3KC3-CI by phosphorylating its components Atg14, AMBRA1 and NRBF2.

**Atg14:** Atg14 is an adaptor protein, which facilitates the recruitment of the PI3KC3 complex to autophagosomes through its targeting sequence BATS (Barkor/Atg14 autophagosome targeting sequence) [72,73]. Atg14 has been shown to mediate the interaction of the PI3KC3-CI with the ULK1 complex through its interaction with Atg13 [45] to promote the recruitment of WIPI2 to phosphatidylinositol phosphates by PI3KC3-CI and LC3 lipidation [74]. Yan et al. first suggested that membrane association of Atg14 or Barkor is negatively regulated by mTORC1 since leucine deprivation induced autophagy by inactivating mTORC1 and facilitating membrane association of Atg14 [75]. Guan and colleagues showed that Atg14 is a direct substrate of mTORC1, which phosphorylates Atg14 at multiple sites including Ser3, Ser223, Thr233, Ser383, and Ser440 (Table 1) [41]. All of these sites were important for mTORC1 to inhibit the PI3KC3-CI activity since mutating any one or all of the sites prevented mTOR-mediated inhibition of PI3KC3 lipid kinase activity [41]. Park et al. reported that ULK1 binds to and phosphorylates Atg14 at Ser29 causing activation of PI3KC3-CI [45]. ULK1-mediated Atg14 phosphorylation was, however, dependent on mTORC1 activity since inhibition/knockdown of mTORC1 increased Ser29 phosphorylation. It is noteworthy that at least one of the mTORC1 phosphorylation sites on Atg14 was part of the BATS domain [72,73,74], and this could potentially be a way for mTORC1 to target the recruitment of PI3KC3-CI to the autophagic membrane.

**AMBRA1:** In basal conditions, AMBRA1, when bound to the PI3K complex via Beclin1, interacts with the dynein light chain 1 and mediates the sequestration of the PI3K complex at the cytoskeleton [69]. Upon phosphorylation on Ser465 and Ser635 by the active ULK1 in nutrient-poor conditions, AMBRA1 detaches from the dynein dock and the PI3KC3 complex translocates to the ER to promote nucleation [69]. The function of AMBRA1 is regulated by mTORC1, which phosphorylates AMBRA1 at Ser52 under normal conditions [44]. Inhibition of mTORC1 results in dephosphorylation of AMBRA1 and allows it to interact with the E3-ligase TRAF6 (tumor necrosis receptor associated factor 6) which ubiquitinates ULK1 on Lys-63 (K63). This K63-linked ubiquitination stabilizes ULK1 by promoting its self-association and further induces its kinase activity [44]. Thus, mTORC1 can negatively regulate autophagy by phosphorylating not only ULK1 but also AMBRA1. 

**NRBF2:** The nuclear receptor binding factor 2 (NRBF2) regulates the lipid kinase activity of the PI3KC3 complex [76]. There are conflicting reports whether NRBF2 is a positive or a negative regulator of autophagy and whether it binds to UVRAG-containing or Atg14-containing PI3KC3 complex [76,77,78]. Ma et al. demonstrated that NRBF2 is a substrate of mTORC1, which phosphorylates human NRBF2 at Ser113 and Ser120 [43] (Table 1). The authors proposed that whether NRBF2 would promote or inhibit autophagy will depend on its phosphorylation status. PI3KC3 can exist in different sub-complexes. When NRBF2 is phosphorylated by mTORC1, it binds to the Vps34-Vps15 non-autophagic complex that has low lipid kinase activity [43]. During nutrient deprivation or when mTORC1 is inactive, dephosphorylated NRBF2 binds to Atg14-Beclin 1 complex to promote PI3KC3 complex assembly, PI3P production, ULK1 association and autophagy induction [43,78]. In NRBF2 knockout MEFs, autophagy is impaired in the nucleation step where the PI3KC3-CI has a more prominent role, as well as in the autophagosome maturation step where the PI3KC3-CII intervenes, suggesting that NRBF2 can interact with either Atg14 or UVRAG [78,79]. Similar to Atg14, NRBF2 interaction with UVRAG could also be key in regulating autolysosome formation [76]. Thus, by phosphorylating NRBF2, mTORC1 has a major handle on a switch for membrane dynamics in autophagy.

#### 3.1.3. Regulation of Autophagosome Expansion

Once PI3P is generated at the omegasome, the next step is the expansion of the phagophore. PI3P recruits WIPI (WD-repeat domain phosphoinositide-interacting protein)-2, the mammalian ortholog of yeast Atg18 and DFCP1 (FYVE domain-containing protein 1) to the omegasome [80,81,82]. The formation of double-membrane autophagosomes requires conjugation of LC3 (mammalian ortholog of yeast Atg8) by the ATG12-Atg5-Atg16L complex. WIPI2 facilitates LC3 lipidation with phosphatidylethanolamine (PE) by binding to Atg16L and recruiting the Atg12-Atg5-Atg16L complex to the phagophore [83]. The Atg12-Atg5-Atg16L complex then becomes part of a conjugation system where the E1-like enzyme Atg7 transfers LC3-I to the E2-like enzyme Atg3 bound to Atg12 on the complex [84,85]. However, deacetylation of LC3 is required for LC3 lipidation and autophagosome formation [47,86]. mTORC1 exerts negative regulation on autophagosome expansion by phosphorylating WIPI2 and p300 acetyltransferase [46,47]. 

**p300:** It is now well established that autophagy is regulated not only by phosphorylation and ubiquitination but also by acetylation. For example, p300 acetyltransferase (p300)-mediated acetylation of PI3KC3/VPS34 suppressed its activity [87]. In addition, acetylation of LC3 regulates shuttling of LC3 between nucleus and cytoplasm [86]. Wan et al. showed that mTORC1 interacts with and directly phosphorylates p300 at Ser2271, Ser2279, Ser2291, and Ser2375 residues at its C-terminal domain [47] (Table 1). This phosphorylation causes activation of p300 by relieving its intra-molecular autoinhibition. Acetylation of LC3 prevents its interaction with E1 ubiquitin ligase Atg7, which activates LC3. During amino acid starvation, dephosphorylation of p300 causes its inactivation and deacetylation of LC3, thereby increasing LC3-Atg7 interaction, LC3 lipidation and autophagosome expansion.

**WIPI2:** Wan et al. recently showed that WIPI2 protein level is regulated by mTORC1 [46]. mTORC1 phosphorylates WIPI2 at Ser395. Phosphorylation of WIPI2 facilitates its interaction with the E3 ubiquitin ligase HUWE1 (HECT, UBA, and WWE Domain Containing 1) and targets it for proteasomal degradation [46]. Thus, inhibition of mTORC1 enhances the stability of WIPI2 and consequently autophagosome formation. This mTORC1-mediated quantity control of WIPI2 affects both basal autophagy and starvation-induced autophagy [46]. 

#### 3.1.4. Regulation of Autophagosome Maturation and Termination

Once the autophagosome formation is complete, autophagosomes fuse with lysosomes to degrade the sequestered materials. The PI3KC3 complex required for autophagosome maturation (PI3KC3-CII) contains UVRAG (UV radiation resistance-associated gene protein) [67], which mediates the interaction of PI3KC3-CII with autophagosome [79] and similar to Atg14, it enhances Vps34 lipid kinase activity [88]. Interaction of UVRAG with the homotypic fusion and vacuole protein sorting (HOPS) stimulates autophagosome-lysosome fusion [79]. UVRAG also interacts with the Run domain protein as Beclin 1 interacting and cysteine-rich containing (Rubicon), which serves as a negative regulator of autophagosome maturation [48,89]. The inhibitory action of Rubicon is antagonized by the protein associated with UVRAG as autophagy enhancer (Pacer), which promotes autophagosome maturation by recruiting Vps34 and the HOPS tethering complex to autophagosomes [90,91]. Autophagic lysosome reformation (ALR), a process by which lysosomal membranes are recycled to maintain lysosome homeostasis during autophagy, marks the last stage of autophagy [92]. This step is needed in order to provide a functional pool of lysosomes for the generation of autolysosomes during prolonged starvation. mTORC1 regulates late stages of autophagy by phosphorylating UVRAG and Pacer (Figure 1 and Table 1).

**UVRAG:** mTORC1 was shown to interact with and phosphorylate UVRAG at Ser498 in nutrient-rich conditions [48]. This phosphorylation enhanced interaction of UVRAG with its negative regulator Rubicon, inhibited the lipid kinase activity of Vps34 and inhibited the interaction between UVRAG and the HOPS complex. Phosphorylation of UVRAG at Ser498 did not affect the initial stages of autophagy, such as the formation of omegasome and phagophore [48]. During starvation, when UVRAG was dephosphorylated, it was released from Rubicon, interacted with HOPS and enhanced autophagosome maturation. 

While inhibition of mTORC1 is needed to induce autophagy so that cellular contents in the lysosomes could be degraded to aid cell survival, reactivation of mTORC1 is required for ALR to maintain lysosome identity. Munson et al. showed that mTORC1 maintains ALR by phosphorylating UVRAG at Ser550 and Ser571, thereby causing activation of Vps34 and generation of PI3P at the lysosome [49]. Loss of phosphorylation at these sites failed to regenerate normal lysosomes and increased cell death during starvation [49]. Thus, mTORC1 regulates the two PI3KC3 complexes differentially. There are several pools of PI3P, including early endosomes, autophagosomes and lysosomes. During nutrient starvation, inhibition of mTORC1 increased autophagic pool of PI3P which is regulated by Atg14-bound PI3KC3-CI but decreased endocytic and lysosomal pool of PI3P which was caused by decrease in UVRAG bound PI3KC3 (PI3KC3-CII) activity [49]. 

**Pacer:** During autophagosome maturation, fusion of the autophagosome membrane with the lysosome membrane requires the HOPS complex, which tethers autophagosomes to lysosomes and syntaxin-17 (Stx-17) to facilitate membrane fusion [93]. Pacer interacts with Stx17 and recruits HOPS to the autophagosomes [91]. Acetylation of Pacer by TIP60 histone acyltransferase facilitates its interaction with the HOPS complex and Stx17 to promote autolysosome formation. Pacer is also a substrate of mTORC1, which phosphorylates Pacer at Ser157 and inhibits its acetylation [50]. During nutrient-rich conditions, mTORC1-mediated phosphorylation of Pacer at Ser157 disrupts its interaction with HOPS and Stx17, preventing autophagosome maturation. During nutrient-deprived conditions, dephosphorylation of Pacer facilitates recruitment of HOPS complex for autophagosome maturation [50].

### 3.2. Transcriptional Regulation of Autophagy by mTORC1

Autophagy is regulated not only by posttranslational modifications of proteins involved in autophagy, but also at the transcriptional level. Several members of the microphthalmia family of basic helix-loop-helix leucine-zipper transcription factors (MiT/TFE), including transcription factor EB (TFEB), TFE3 and MITF have been shown to play important roles in lysosome biogenesis and autophagy [94]. 

A systems biology study identified a transcriptional network of genes involved in lysosome biogenesis and TFEB as a master regulator of autophagy [95]. TFEB upregulates a subset of genes required for autophagosome formation, fusion of autophagosomes with lysosomes and lysosome biogenesis by binding to the Coordinated Lysosomal Expression and Regulation (CLEAR) elements present in the promoter regions of these genes. Overexpression of TFEB was shown to increase the expression of UVRAG, WIPI, MAPLC3B, SQSTM1, VPS11, VPS19, and ATG9B that are involved in various steps of autophagy [96]. TFE3 was also shown to regulate autophagy and lysosome homeostasis in a manner similar to TFEB [97,98]. During starvation, dephosphorylation of TFE3 resulted in its translocation to the nucleus where it induced expression of genes, such as ATG16L1, ATG9B, GABARAPL1, and WIPI1 for the formation of autophagosomes, and UVRAG for the fusion of autophagosomes with lysosomes.

The activity of TFEB/TFE3 depends on their subcellular localization, which in turn is determined by the nutrient availability as well as their phosphorylation status. TFEB contains a nuclear localization sequence (NLS) [99] and a nuclear export signal (NES) [100], and shuttles between the nucleus and the cytoplasm. These sites are also conserved in TFE3 [99]. Several studies showed that TFEB and TFE3 are substrates of mTORC1. During nutrient-rich conditions when amino acids are abundant, Rag-GTPases sense the amino acid status via V-ATPase and recruits mTORC1 to the lysosomes to activate it. Active Rag GTPases can also bind to and recruit TFEB/TFE3 on the surface of lysosomes [101] where mTORC1 phosphorylates TFEB at Ser211 [97,102], Ser122 [103] and Ser142 [101], and TFE3 at Ser321 [100]. It has been proposed that phosphorylation of TFEB at Ser211 masks its NLS and creates a binding site for the 14-3-3 family of proteins, which sequester TFEB and retain it in the cytoplasm [97,102]. Nutrient deprivation or inactivation of mTORC1 results in the loss of mTORC1 from the lysosome. Dephosphorylation of TFEB/TFE3 by PP2A also results in their activation independent of mTORC1 inhibition [104]. When TFEB/TFE3 are no longer phosphorylated at Ser211 or Ser321, they dissociate from 14-3-3 proteins and translocate to the nucleus to turn on gene expression [98]. 

Subsequently, it has been shown that phosphorylation of TFEB at Ser211 was not sufficient to exclude it completely from the nucleus since mutation of Ser211 to Ala caused diffused localization of TFEB in both the nucleus and the cytoplasm, suggesting additional mechanism(s) contributing to nucleo-cytoplasmic shuttling of TFEB [103]. mTORC1 could phosphorylate TFEB in vitro at Ser122 but phosphorylation of TFEB at Ser122 alone was not sufficient to retain TFEB in the cytosol. However, phospho-mimicking mutant of TFEB (Ser122 to Asp) prevented nuclear transport of TFEB following inhibition of mTORC1. It was proposed that while phosphorylation at Ser211 is responsible for promoting interaction of TFEB with 14-3-3 proteins and its cytosolic retention, phosphorylation at Ser122 is also needed for nuclear transport of TFEB via protein-protein interaction or some other as yet unidentified mechanism [103]. 

Recent studies showed that TFEB interacts with the major exportin chromosome region maintenance 1 (CRM1), which can export TFEB out of the nucleus [100,105]. mTORC1-mediated phosphorylation of TFEB was also responsible for its nuclear export. While Napolitano et al. reported that mTORC1 phosphorylates TFEB at both Ser138 and Ser142 sites located near the NES [100], Li et al. reported that phosphorylation of TFEB on Ser142 by mTORC1 primes its phosphorylation on Ser138 by glycogen synthase kinase-3-β (GSK3β), thereby permitting CRM1-mediated nuclear export of TFEB; phosphorylation at both sites was required for the nuclear export of TFEB [105]. Interestingly, the inactivating phosphorylation of GSK3β itself is mediated by the mTORC2/Akt and not mTORC1. During glucose starvation, GSK3β phosphorylation was inhibited by Torin 1 and Rictor knockdown but not by rapamycin [105]. Thus, phosphorylation of TFEB by mTORC1 at multiple sites is needed to retain it in the cytosol and to ensure its complete inactivation, and both mTORC1 and mTORC2 regulate nuclear export of TFEB. Phosphorylation of TFEB at Ser142 and Ser211 may also target TFEB for ubiquitin-mediated degradation [106], suggesting that mTORC1 may regulate both the subcellular localization as well as the stability of TFEB.

In contrast to aforementioned studies, one report suggested that phosphorylation of TFEB by mTORC1 promotes its translocation to the nucleus and increases transcription of its target gene V-ATPase [107]. While the reason for this discrepancy is not clear, it is worth mentioning a few differences in the experimental setup that may influence the outcome from these studies. First, when mTORC1 was activated by depleting TSC2, TFEB phosphorylation induced its nuclear translocation, which could be blocked by mTORC1 inhibitor rapamycin [107]. When experiments were performed with TSC2-positive cells, phosphorylation of TFEB prevented its nuclear translocation [96,97,102,108], which could be blocked by mTORC1/2 inhibitor Torin 1 but not by rapamycin [108]. Second, inhibitors of PI3K and Akt were more effective than rapamycin in inducing nuclear translocation of TFEB [102]. Finally, TFEB-induced gene expression was distinct. While Settembre et al. reported induction of genes involved in autophagy and lysosome biogenesis following TFEB overexpression [96], Pena-Llopis et al. reported induction of v-ATPase and enrichment of genes associated with glycolysis, pentose phosphate pathway and fatty acid biosynthesis [107]. It is important to consider that mTORC1 is activated downstream of PI3K/Akt which inhibits TSC2 in growth factor-dependent pathway whereas amino acids activate mTORC1 independent of Akt/TSC2 pathway. Given that nuclear translocation was inhibited more effectively by PI3K/Akt inhibitors compared to rapamycin [102], the contribution of this arm of mTORC1 should not be ignored. Alternatively, since Akt acts downstream of mTORC2, and Torin 1 but not rapamycin inhibits mTORC2, the contribution of mTORC2 should also be considered. There is also a cross-talk between mTORC1 and mTORC2. Finally, nucleo-cytoplasmic shuttling of TFEB is a dynamic process and the timing of mTORC1 inhibition may influence the outcome.

A recent study showed that the Microphthalmia-associated transcription factor (MITF), another member of the MiT/TFE family, could induce autophagy via upregulation of microRNA 211 (*MIR211*), which induced nuclear translocation of MITF [109]. Torin 1, which inhibits both mTORC1 and -2, induced nuclear translocation of MITF. The mechanism by which MITF regulates autophagy involves cross-talk between mTORC2 and mTORC1. MITF suppressed the expression of mTORC2 binding partner Rictor thereby causing inhibition of its downstream target Akt. Since Akt acts upstream of mTORC1, inhibition of Akt results in the inhibition of mTORC1 to promote nuclear translocation of MITF and triggers expression of genes involved in autophagy, such as LC3B, ATG16L1, SQSTM1, ATG9B, and UVRAG [109]. Thus, like TFEB and TFE3, inhibition of mTORC1 was also responsible for nuclear translocation of MITF. 

While mTORC1-mediated phosphorylation can alter the subcellular localization, stability and activity of the MiT/TFE family of transcription factors, these transcription factors may, in turn, regulate mTORC1 activity. The tumor suppressor folliculin (FLCN), which plays an important role in nutrient sensing, is a transcriptional target of TFEB/TFE3 [110]. During starvation, FLCN is upregulated by TFE3/TFEB and is recruited to lysosomal membranes where it causes reactivation of mTORC1, thus allowing phosphorylation and retention of TFE3 in the cytosol [98]. During starvation, the activation of MiTF transcription factors TFEB, TFE3 and MITF triggers transcription of *RagD* mRNA. Thus, an increase in RagD results in mTORC1 activation as part of a feedback loop [111]. 

While inhibition of mTORC1 is needed for the initiation of autophagy, reactivation of mTORC1 is required for the termination of autophagy. During the final stage, autophagosomes fuse with lysosomes to degrade autolysosomal contents [112]. During prolonged starvation, replenishment of lysosomes is needed to maintain lysosome homeostasis/function in order to sustain autophagy [112]. Nnah et al. showed that TFEB induces genes required for the formation of endocytic vesicles which carry components of the lysosomal nutrient sensing complex (LYNUS) required for activating mTORC1 and tethers mTORC1 to lysosomal membranes. [113]. LYNUS complex contains p-Akt, which phosphorylates and releases TSC2 and the Ras related GTPase binding D (*RRAGD*)/RagD, which tethers mTORC1 to endolysosomes causing its activation. 

SQSTM1, which encodes p62, a scaffold protein that binds ubiquitin-tagged autophagy substrates for their sequestration in the autophagosome, is also a transcriptional target of these MiT/TFE transcription factors [96,109,114]. p62 plays an important role in nutrient sensing [115]. When amino acids are abundant, p62 interacts with Raptor and facilitates the interaction of the mTORC1 complex with Rag proteins on the lysosomal membrane [115]. p62 also interacts with the E3 ubiquitin ligase TRAF6 and recruits it to the lysosomes where K63-linked polyubiquitination of mTORC1 results in its activation [116]. Thus, this dual ability of the MiT/TFE transcription factors to induce autophagy-enabling genes or to promote mTORC1 activation based on the need of the cell, places them in the “fulcrum” position of a scale, ensuring the balance between autophagy and cell survival.

## 4. Conclusions

At the crossroads of physiological and pathological processes, autophagy paves the way for the redistribution of cellular resources to where they are most needed, and it comes with intricate regulation, even more so due to its importance in ensuring cell survival. As a major regulator of cell metabolism, mTOR signaling is at the forefront of autophagy regulation. Owing to the fact that nutrient availability and cellular stress modulate mTORC1 activity, mTORC1 is a consistent inhibitor of autophagy. mTORC1 is able to perform this role by casting a wide net on autophagy and targeting each step of the autophagy process via the downstream targets. As discussed in this review, experimental settings may greatly influence the interpretation, and may contribute to contradictory findings. It is important to consider the timing of autophagy (acute versus prolonged), media composition (amino acid starvation, glucose deprivation, the type of amino acid in the media, such as Gln, Leu, Arg, and the presence of EBSS or serum) and the trigger of autophagy. Another important consideration is the dynamic nature of protein-protein interactions and localization of mTORC1 and its targets. Several kinases besides mTORC1 are involved in the regulation of autophagy including AMPK, Akt, PKC, and MAPK amongst others, but the cross-talk and feedback regulation of mTORC1 with these kinases may also influence autophagy [117,118]. Emerging studies suggest that mTORC2 may also regulate autophagy [105,109]. This may explain why in some studies Torin 1, which inhibits both mTORC1 and mTORC2, but not rapamycin, which inhibits primarily mTORC1, exerted an effect [105,109]. In addition, some of the downstream targets of mTORC1, such as 4E-BP1 is less sensitive to inhibition by rapamycin [119]. Since pharmacological inhibitors may lack specificity, it is important to consider proper molecular approaches, such as RNA interference or CRISPR/Cas 9 knockouts to interpret the data. A thorough understanding of how mTOR signaling regulates different steps of autophagy is essential to exploit this master regulator of autophagy to treat human diseases.

## Figures and Tables

**Table 1:** Phosphorylation of mTORC1 targets at various steps of autophagy.

Step of Autophagy	mTORC1 Substrate	Phosphorylation Sites	Regulation	References
**Induction**	**ULK1**	Ser637 (Ser638) Ser757 (Ser758)	Inhibits ULK1 kinase activity and modulates interaction of ULK1 with AMPK	[48,55]
	**Atg13**	Ser258	Inhibits ULK1 kinase activity	[56]
**Nucleation**	**Atg14**	Ser3 Ser383 Ser440 Thr233	Inhibits Vps34 lipid kinase activity of the PI3KC3 complex I	[41]
	**AMBRA1**	Ser52	Docks the PI3KC3 complex I at the cytoskeleton and inhibits its recruitment at the ER	[44]
	**NRBF2**	Ser113 Ser120	Increases the binding affinity of NRBF2 for Vps34-Vps15 which inhibits the lipid kinase activity of the PI3KC3 complex I	[43]
**Autophagosome elongation**	**WIPI2**	Ser395	Promotes binding of WIPI2 with the E3 ligase HUWE1 and its subsequent degradation	[46]
	**P300**	Ser2271 Ser2279 Ser2291 Ser2375	Inhibits the autoinhibition of p300 and promotes the acetylation of LC3 which impedes the lipidation of LC3	[47]
**Autophagosome maturation**	**UVRAG**	Ser498	Enhances the affinity of UVRAG for RUBICON which inhibits the recruitment of the HOPS tethering complex	[48]
		Ser550 Ser571	Promotes the activity of UVRAG-Vps34 towards lysosomal tubulation	[49]
	**Pacer**	Ser157	Inhibits the acetylation of Pacer by TIP60 which promotes the binding of Pacer to UVRAG and the recruitment of the HOPS tethering complex	[50]
